# Multivariate analytical approaches for investigating brain-behavior relationships

**DOI:** 10.3389/fnins.2023.1175690

**Published:** 2023-07-31

**Authors:** E. Leighton Durham, Karam Ghanem, Andrew J. Stier, Carlos Cardenas-Iniguez, Gabrielle E. Reimann, Hee Jung Jeong, Randolph M. Dupont, Xiaoyu Dong, Tyler M. Moore, Marc G. Berman, Benjamin B. Lahey, Danilo Bzdok, Antonia N. Kaczkurkin

**Affiliations:** ^1^Department of Psychology, Vanderbilt University, Nashville, TN, United States; ^2^Department of Biomedical Engineering, McGill University, Montreal, QC, Canada; ^3^Department of Psychology, University of Chicago, Chicago, IL, United States; ^4^Department of Population and Public Health Sciences, Keck School of Medicine, University of Southern California, Los Angeles, CA, United States; ^5^Department of Psychiatry, Perelman School of Medicine, University of Pennsylvania, Philadelphia, PA, United States; ^6^The University of Chicago Neuroscience Institute, University of Chicago, Chicago, IL, United States; ^7^Department of Health Studies, University of Chicago, Chicago, IL, United States; ^8^Department of Psychiatry and Behavioral Neuroscience, University of Chicago, Chicago, IL, United States

**Keywords:** canonical correlation analysis, partial least squares, gray matter volume, psychopathology, brain development

## Abstract

**Background:**

Many studies of brain-behavior relationships rely on univariate approaches where each variable of interest is tested independently, which does not allow for the simultaneous investigation of multiple correlated variables. Alternatively, multivariate approaches allow for examining relationships between psychopathology and neural substrates simultaneously. There are multiple multivariate methods to choose from that each have assumptions which can affect the results; however, many studies employ one method without a clear justification for its selection. Additionally, there are few studies illustrating how differences between methods manifest in examining brain-behavior relationships. The purpose of this study was to exemplify how the choice of multivariate approach can change brain-behavior interpretations.

**Method:**

We used data from 9,027 9- to 10-year-old children from the Adolescent Brain Cognitive Development^SM^ Study (ABCD Study^®^) to examine brain-behavior relationships with three commonly used multivariate approaches: canonical correlation analysis (CCA), partial least squares correlation (PLSC), and partial least squares regression (PLSR). We examined the associations between psychopathology dimensions including general psychopathology, attention-deficit/hyperactivity symptoms, conduct problems, and internalizing symptoms with regional brain volumes.

**Results:**

The results of CCA, PLSC, and PLSR showed both consistencies and differences in the relationship between psychopathology symptoms and brain structure. The leading significant component yielded by each method demonstrated similar patterns of associations between regional brain volumes and psychopathology symptoms. However, the additional significant components yielded by each method demonstrated differential brain-behavior patterns that were not consistent across methods.

**Conclusion:**

Here we show that CCA, PLSC, and PLSR yield slightly different interpretations regarding the relationship between child psychopathology and brain volume. In demonstrating the divergence between these approaches, we exemplify the importance of carefully considering the method’s underlying assumptions when choosing a multivariate approach to delineate brain-behavior relationships.

## 1. Introduction

Studies investigating relationships between psychopathology and the brain have typically taken a univariate approach where each variable of interest is tested independently (Bzdok, 2017; Snyder et al., 2017; Romer et al., 2018; Bzdok and Ioannidis, 2019; Kaczkurkin et al., 2019; Moore et al., 2019; Durham et al., 2021). However, neither psychopathology symptoms nor brain regions are independent from one another. Multivariate analytical approaches attempt to address this by allowing for the simultaneous examination of multiple correlated variables in one model (McIntosh and Mišić, 2013; Xia et al., 2018; Kaczkurkin et al., 2020; Wang et al., 2020; Zhuang et al., 2020). The most commonly employed multivariate analytical approaches in the study of brain-behavior relationships are canonical correlation analysis (CCA), partial least squares correlation (PLSC) analysis, and partial least squares regression (PLSR) analysis. The primary difference between CCA and PLS methods is whether the approach aims to maximize the correlation (CCA) or the covariance (PLS) between variable sets (McIntosh and Mišić, 2013). Further, the primary difference between PLSC and PLSR is that PLSC is designed to identify *associations* between two sets of data while PLSR creates latent variables from one dataset to *predict* the values of another dataset (Krishnan et al., 2011). Multivariate methods such as CCA, PLSC, and PLSR can better capture brain-behavior relationships by directly accounting for the complex interrelationships between all variables in a single model estimation. A growing body of literature has employed these multivariate techniques to the study of psychopathology and neuroscience (Berman et al., 2014; Lin et al., 2018; Moser et al., 2018; Rodrigue et al., 2018; Stout et al., 2018; Kebets et al., 2019; Mihalik et al., 2019; Supekar et al., 2019; Kaczkurkin et al., 2020; Wang et al., 2020).

Despite the differences between these three multivariate methods, much of the work around brain-behavior relationships using such approaches utilizes one method or another without an evident justification driving method selection, despite their conceptual and statistical differences. In CCA, the goal is to find a pair of linear transformations, one representing each set of variables, such that the variables are maximally correlated in the transformed (embedding) space (Hotelling, 1992). In PLSC, two datasets are correlated by identifying which data co-occurs and finding pairs of latent vectors with maximal covariance (Krishnan et al., 2011). Finally, PLSR, the only clearly predictive method among the three, fits a linear regression model by projecting each set of variables into a latent space and maximizing the covariance structures in these two spaces (Wold et al., 2001; Krishnan et al., 2011). Additionally, only CCA methods standardize the covariance matrix (i.e., whitening) across datasets, while PLS methods do not routinely apply this data preprocessing step (Yu and MacGregor, 2004; Jendoubi and Strimmer, 2019). Given the distinct properties of these different statistical tools, it is important to carefully consider which method is most appropriate to address the research question and study context at hand.

The aim of the current study is to demonstrate the differences in the interpretations of results across these three multivariate analytical approaches when applied to the study of brain-behavior relationships in children. To accomplish this, associations between 87 regional gray matter volumes (GMV) and four dimensions of psychopathology (general psychopathology, internalizing symptoms, ADHD symptoms, and conduct problems) were investigated using CCA, PLSC, and PLSR. Data for these analyses came from a large sample (*N* = 9,027) of 9- to 10-year-old children from the Adolescent Brain Cognitive Development^SM^ Study (ABCD Study®). The present analyses exemplify how the differences between alternative multivariate analytical approaches may influence research findings and their corresponding interpretations in the context of studying brain-behavior relationships.

## 2. Materials and methods

### 2.1. Participants

The current study used data from Wave 1 (release 4.0) of the Adolescent Brain Cognitive Development (ABCD) Study (Volkow et al., 2018), which includes data from 11,876 children from 9 to 10 years of age. The use of this dataset was approved by the Vanderbilt University institutional review board. Recruitment strategies for the ABCD Study are detailed elsewhere (Garavan et al., 2018) and summarized in the supplement. For the present analyses, participants were excluded if they had missing data on variables included in the analysis or for failure to pass data quality assurance measures (*N* = 1,184) (see Supplementary material for more details). An additional 1,665 same-family participants were randomly excluded to control for non-independence between twins and siblings. The final sample size used for analysis was *N* = 9,027. A summary of the demographic characteristics of the sample can be found in Table 1.

**Table 1:** Summary of demographic characteristics of the sample for analyses of multivariate associations between brain volume and psychopathology (*N* = 9,027).

		*Mean*	*SD*
Age (months)		118.92	7.41
		*N*	*%*
*Gender*			
	Female	4,317	47.82
	Male	4,710	52.18
*Race-ethnicity*			
	Non-Hispanic White	4,627	51.26
	Hispanic	1,932	21.40
	Black/African American	1,336	14.80
	Other	1,132	12.54
*Household annual income*			
	< $5,000	311	3.44
	$5,000–$11,999	332	3.68
	$12,000–$15,999	228	2.52
	$16,000–$24,999	398	4.41
	$25,000–$34,999	510	5.65
	$35,000–$49,999	694	7.69
	$50,000–$74,999	1,129	12.51
	$75,000–$99,999	1,215	13.46
	$100,000–$199,999	2,496	27.65
	≥$200,000	937	10.38
	Missing	777	8.61
*Parental education*			
	No degree	473	5.24
	High school degree/GED	1,116	12.36
	Some college	1,471	16.30
	Associate’s degree	1,165	12.91
	Bachelor’s degree	2,516	27.87
	Master’s degree	1,737	19.24
	Professional/Doctoral degree	549	6.08

The Race-Ethnicity category “Other” includes children identified by their parent as American Indian/Native American, Alaska Native, Native Hawaiian, Guamanian, Samoan, Other Pacific Islander, Asian Indian, Chinese, Filipino, Japanese, Korean, Vietnamese, Other Asian, Multi-Racial or Other Race.

### 2.2. Measure of psychopathology

Psychopathology symptoms were assessed with the Child Behavior Checklist (CBCL) for school-aged children (Achenbach, 2009), which was completed by one parent or guardian of each participant. The CBCL consists of 119 items that describe emotional and behavioral problems rated with a 3-point scale [0 = *not true (as far as you know)*, 1 = *somewhat or sometimes true*, and 2 = *very true or often true*]. The CBCL items that were included in the current study demonstrated strong internal consistency in the present sample (*α* = 0.94).

### 2.3. Hierarchical modeling of psychopathology dimensions

Psychopathology symptoms assessed with the CBCL were hierarchically modeled for the purpose of analysis. This was done to allow for investigations of multivariate associations between brain volume and both general and specific dimensions of psychopathology. The hierarchical modeling procedures used for the present analyses are described in detail in our prior work (Moore et al., 2020). Psychopathology factors were modeled using Mplus version 8.4.^1^ First, exploratory structural equation modeling (ESEM) was used to identify latent factors from 66 CBCL items, as was done previously (Moore et al., 2020). The exploratory analysis yielded 3 correlated dimensions of psychopathology (internalizing, ADHD, and conduct problems). A confirmatory bifactor analysis was then used to model these three symptom dimensions plus a general psychopathology factor that reflects the shared symptoms across all domains. All factors are orthogonal to each other (Lahey et al., 2017). These factors met recommended standards for factor determinacy and construct reliability and showed adequate criterion validity (Moore et al., 2020). Additional details can be found in the original manuscript by Moore et al. (2020) and in the supplement.

### 2.4. Image acquisition, processing, and quality assurance

Procedures for image acquisition, image processing, and quality assurance have been described previously (Casey et al., 2018) and are summarized in the supplement. Briefly, 3 T MRI imaging occurred at all study sites and the ABCD Data Analysis and Informatic Center (DAIC) completed centralized processing and analysis procedures. Cortical surface reconstruction and subcortical segmentation were performed based on automated, atlas-based, segmentation procedures in FreeSurfer v5.3 (Fischl, 2012). The present analyses included 68 cortical regions that were derived from the surface-based atlas procedure developed by Desikan et al. (2006) in addition to 19 subcortical regions that were derived by the automated labeling procedure developed by Fischl et al. (2002).

### 2.5. Statistical analysis

**Figure 1:**
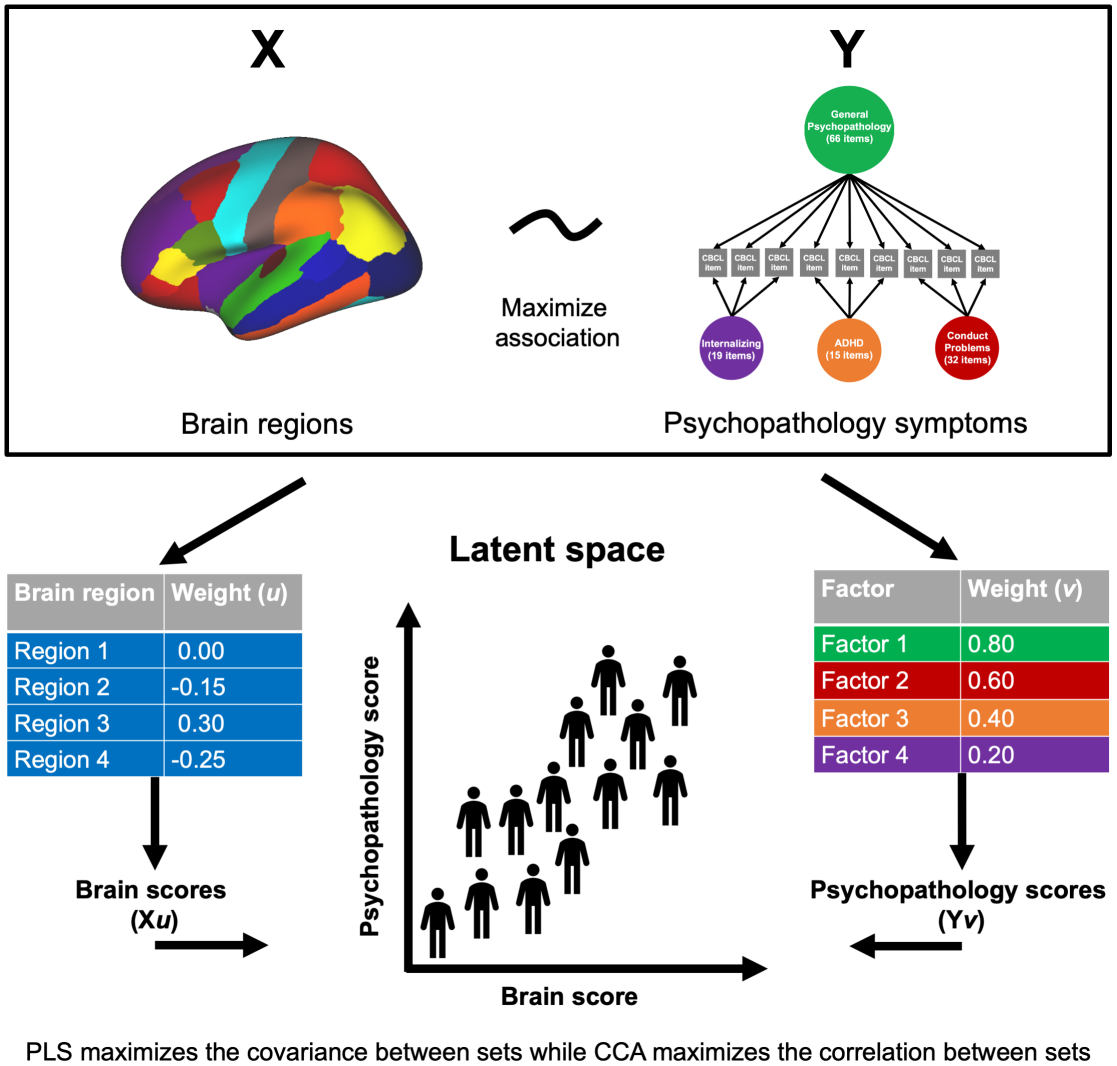
Multivariate approaches to understanding brain-behavior relationships. Two sets of variables, *X* and *Y* (in this case, brain volume and psychopathology symptoms), serve as the input data into the multivariate approach. Weight vectors (*u* and *v*) are identified that maximize the covariance or correlation between linear combinations of the brain and psychopathology variables. Brain scores for each individual are made by weighting the participant’s brain volume by the corresponding weight (*Xu*), and likewise for psychopathology variables (*Yv*). This yields a weighted score for each participant on each set of variables that can then be projected to a latent space to examine brain-behavior relationships.

Using the dimensions of psychopathology originally identified by Moore et al. (2020), we examined associations between psychopathology and GMV by adopting a sequence of different multivariate models: CCA, PLSC, and PLSR (Figure 1). In the current study, a set of 87 regional gray matter volumes (68 cortical and 19 subcortical) and a set of the four psychopathology dimensions (general psychopathology, conduct problems, ADHD, and internalizing) provided the input for our analyses. Variance inflation factor values are presented for all variables in Supplementary Table S1. The CCA, PLSC, and PLSR were performed using the scikit-learn program in Python.^2^ The two PLS approaches (PLSC and PLSR) maximize the covariance across variable sets (McIntosh and Mišić, 2013) and thus, consider the whole covariance structure. PLSC is a correlational approach within that framework, while PLSR is a predictive approach (Krishnan et al., 2011). Alternatively, the CCA approach maximizes the correlations across the latent embeddings of the two variable sets and disregards the auto-correlation structure within each variable set, essentially using whitened data (Jendoubi and Strimmer, 2019). Thus, CCA linearly removes the covariance between brain regions and assumes that larger GMV in a certain region in an individual is completely independent from all other brain regions in the same individual. Using all three approaches, we examined how the relationships between GMV and psychopathology in children may differ or converge based on the method used. In each approach, permutation testing with 1,000 iterations was conducted to test the significance of four latent variables yielded by each analysis type. A component with a significant *p*-value suggests a strong coupling of associations between the psychopathology dimensions and regional brain volumes. The structure coefficient (*r_s_*), or the bivariate correlation between an observed variable and a synthetic variable, is also reported for each significant component. Age, sex, race/ethnicity, and MRI manufacturer were treated as covariates in all analyses and were regressed out of the brain and psychopathology variables prior to applying the multivariate methods. Input variables were standardized prior to analyses. Sensitivity analyses were performed with intracranial volume (ICV) added as an additional covariate.

### 2.6. Comparison between models

To quantify each model’s statistical performance, we evaluated the explained variance for all the derived unique components of each method and found the significance of each method’s components using their calculated Pearson rho values. Through permutation analyses with 1,000 permutations, the significance of the correlation coefficients obtained from the components of the model in question are assessed by generating randomized permuted versions of the psychopathology factors, fitting the model with the resultant permuted versions of the psychopathology factors and the brain volume data, and then calculating the correlation coefficients and model scores of each component. The results are then compared to the original observed values from the original version of the model fitted with unpermuted data to compute *p*-values. The *p*-values are used to present the resulting significance of each component in every model, which helps create an apple-to-apple statistical comparison of the performance of each model.

## 3. Results

### 3.1. Canonical correlation analysis results

**Figure 2:**
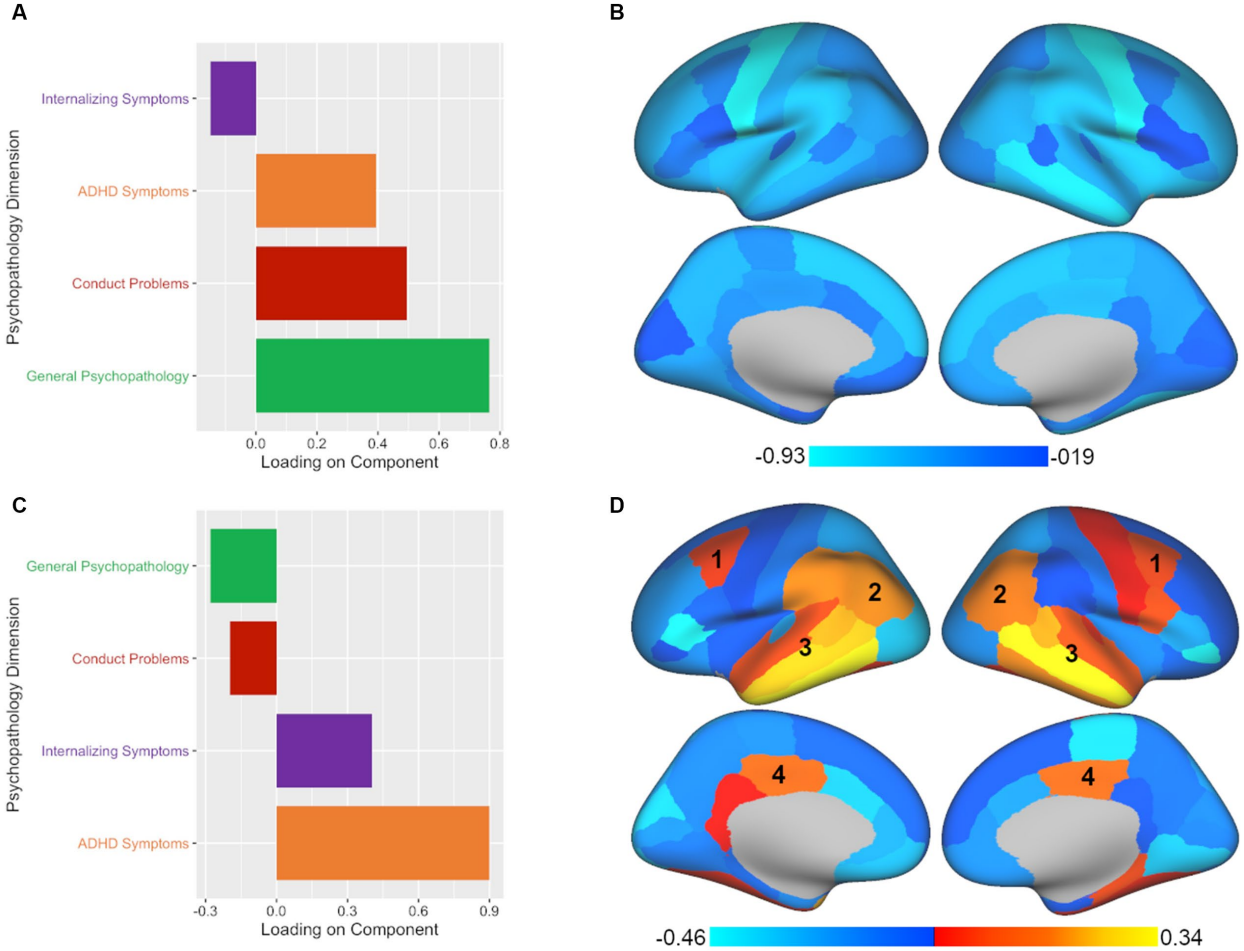
CCA analysis yields two significant components illustrating associated patterns of psychopathology and brain volume. All plotted values represent component loadings. **(A)** The leading significant component yielded by CCA included mostly general psychopathology symptoms with conduct problems and ADHD symptoms making secondary contributions. **(B)** The psychopathology pattern in panel A was associated with a relatively global pattern of regional volumes across the brain. **(C)** The second significant component yielded by CCA extracted primarily ADHD symptoms with internalizing symptoms making a secondary contribution. **(D)** The psychopathology pattern in panel C was associated with a mixed pattern of regional volumes across the brain. Regions which showed commonalities across the second significant components of all three multivariate approaches included the (1) caudal middle frontal, (2) inferior parietal, (3) superior, middle, and inferior temporal, and (4) posterior cingulate regions.

Of the four components tested, CCA yielded two significant components, with each representing different aspects of the relationship between GMV and psychopathology in children. The leading significant component (*p* < 0.001, *r_s_* = 0.159) primarily highlighted general psychopathology symptoms with conduct problems and ADHD symptoms making secondary contributions (see Figure 2; Supplementary Table S2). In terms of brain volume, the leading component was associated with a relatively global pattern across the brain (see Figure 2; Supplementary Table S3). The second component (*p* = 0.03, *r_s_* = 0.111) primarily extracted ADHD symptoms with internalizing symptoms making a secondary contribution (see Figure 2; Supplementary Table S2). For brain volume, the second component was associated with a mixed pattern across regional volumes in terms of direction of association (see Figure 2; Supplementary Table S3). When adding ICV as an additional covariate, we found the results to be largely consistent with the primary results both in terms of number of significant components and in the brain-behavior relationships demonstrated.

### 3.2. Partial least squares correlation analysis results

**Figure 3:**
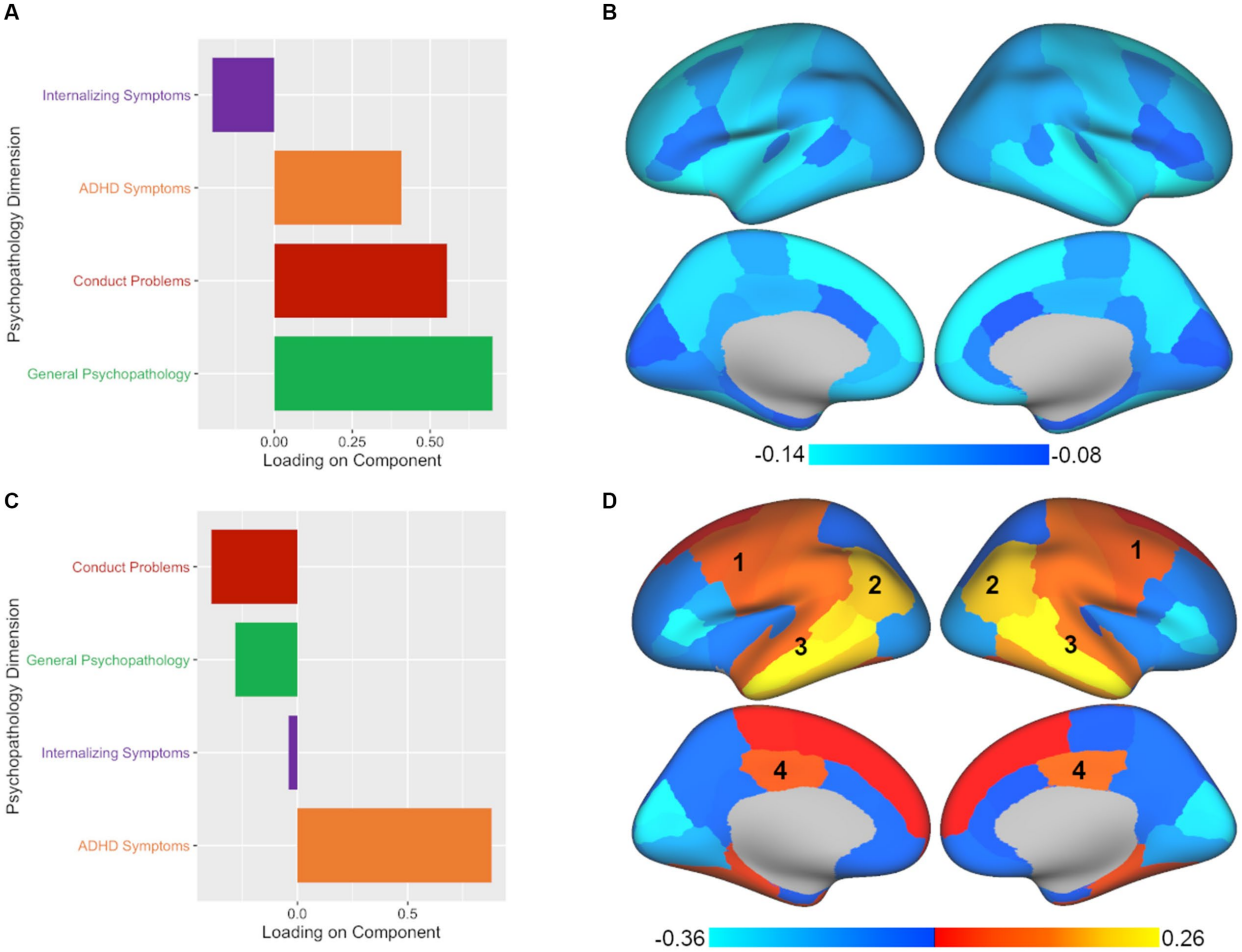
PLSC analysis yields two significant components with different associations between patterns of psychopathology and brain volume. Plotted values represent component loadings. **(A,B)** The psychopathology pattern in panel A and the brain regions in panel B found for PLSC’s leading component are nearly identical to those found in CCA, with slight variations in the intensity of the brain region results. **(C,D)** The second significant component yielded by PLSC diverged from CCA by primarily extracting ADHD symptoms and not internalizing symptoms, while some commonalities in brain regions continued to be apparent in the (1) caudal middle frontal, (2) inferior parietal, (3) superior, middle, and inferior temporal, and (4) posterior cingulate regions. At the same time, additional brain regions in the PLSC second component results show an opposite pattern to those found in the CCA second component.

PLSC analysis also yielded two significant components of the four components tested. The leading significant component (*p* < 0.001, *r_s_* = 0.104) was again predominantly comprised of general psychopathology symptoms with secondary contributions by conduct problems and ADHD symptoms (see Figure 3; Supplementary Table S4). In terms of brain volume, the leading component was associated with a relatively global pattern across the brain, similar to the leading component found in CCA (see Figure 3; Supplementary Table S5). However, unlike the CCA results, the second component in PLSC (*p* < 0.05, *r_s_* = 0.084) was dominated by ADHD symptoms (see Figure 3; Supplementary Table S4) without much of any contribution from internalizing symptoms. Despite the difference in the representation of psychopathology symptoms between CCA and PLSC for the second component, in terms of brain volume, there were commonalities between the CCA and PLSC results for regions such as the caudal middle frontal, inferior parietal, superior temporal, middle temporal, inferior temporal, and posterior cingulate (see Figure 3; Supplementary Table S5). As can be seen in Figures 2, 3, PLSC also found brain associations in opposite directions than CCA. After adding ICV as an additional covariate, the results were largely consistent with the primary results both in terms of number of significant components and in the brain-behavior relationships observed.

### 3.3. Partial least squares regression analysis results

**Figure 4:**
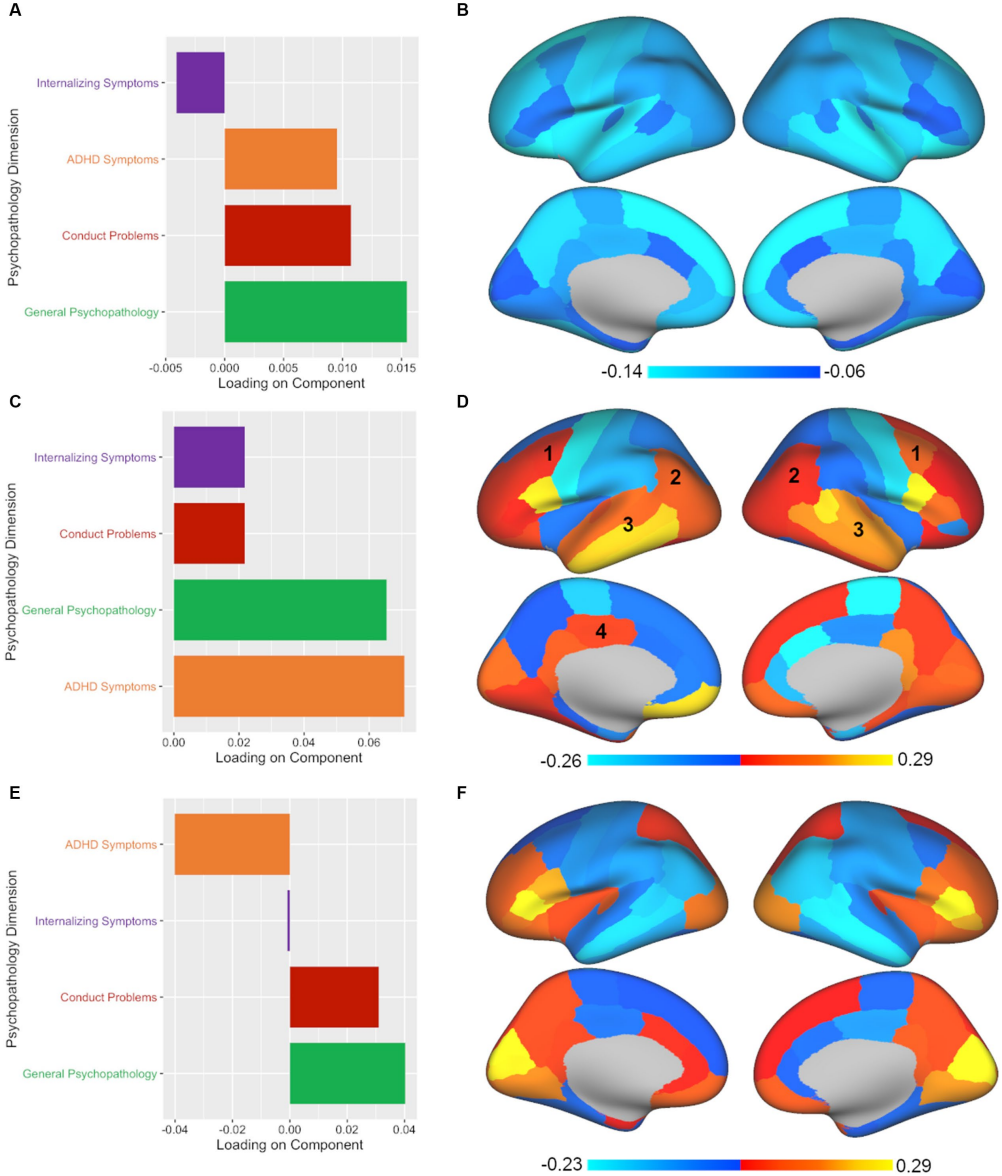
PLSR analysis shows divergent results from CCA and PLSC, yielding three significant components with both common and unique associations between psychopathology and brain volume. Plotted values in all panels represent component loadings. **(A,B)** Again, an almost identical pattern (mostly general psychopathology, some conduct problems and ADHD symptoms) was associated with a global pattern across the brain in PLSR, as was also found in CCA and PLSC. **(C)** PLSR also pulled out a second significant component, but unlike CCA’s second component (which extracted ADHD and internalizing symptoms) or PLSC’s second component (which found ADHD symptoms alone), the second component in PLSR coupled ADHD symptoms and general psychopathology symptoms. **(D)** Again, a mixed pattern of regional volumes across the brain were found for the second component with some of the same common regions implicated: (1) caudal middle frontal, (2) inferior parietal, (3) superior, middle, and inferior temporal, and (4) posterior cingulate (although the posterior cingulate was unilateral in PLSR). **(E)** PLSR was the only multivariate method tested to extract a third component. The third component yielded by PLSR was predominantly influenced by general psychopathology symptoms with conduct problems making a secondary contribution while ADHD symptoms showed an inverse pattern. **(F)** The brain regions implicated in the third component in PLSR showed unique associations with frontal and lateral occipital regions.

Finally, PLSR analysis yielded three significant components out of the four components tested. The leading significant component (*p* < 0.001, *r_s_* = 0.104) was again associated with primarily general psychopathology symptoms with secondary contributions from conduct problems and ADHD symptoms (see Figure 4; Supplementary Table S6). As with CCA and PLSC, PLSR also showed a relatively global pattern across the brain (see Figure 4; Supplementary Table S7). The second component in PLSR (*p* < 0.001, *r_s_* = 0.096) diverged from both CCA and PLSC by extracting ADHD symptoms and general psychopathology symptoms as the primary contributors (see Figure 4; Supplementary Table S6). Commonalities that continued to be apparent in terms of brain volume for the second component of PLSR included caudal middle frontal, inferior parietal, superior temporal, middle temporal, inferior temporal, and posterior cingulate regions (see Figure 4; Supplementary Table S7), although the posterior cingulate finding was not bilateral in PLSR. In contrast to both CCA and PLSC, PLSR was the only multivariate method to extract a third component. The third component (*p* < 0.05, *r_s_* = 0.083) consisted primarily of general psychopathology symptoms and some conduct problems, with ADHD contributing in the opposite direction (see Figure 4; Supplementary Table S6). In terms of brain volume, the third component of PLSR was associated with some common regions with the other two methods, but also some unique findings, such as frontal regions and the lateral occipital region (see Figure 4; Supplementary Table S7). Finally, adding ICV resulted in largely consistent results both in terms of number of significant components and in the illustrated brain-behavior relationships.

## 4. Discussion

The current study examined the commonalities and divergence in brain-behavior relationships derived from three different multivariate statistical approaches (CCA, PLSC, and PLSR) in a large sample of children (*N* = 9,027). The results of CCA, PLSC, and PLSR were characterized by both consistencies and differences across associated patterns of psychopathology symptoms and regional brain volumes. The leading significant component yielded by each method demonstrated similar patterns of associations between regional brain volumes and psychopathology symptoms. However, the additional significant components yielded by each method demonstrated differential brain-behavior patterns that were not entirely consistent across methods.

All three methods yielded a similar leading component suggesting a common pattern of psychopathology symptoms and associated brain volumes. In particular, the leading component in CCA, PLSC, and PLSR each extracted general psychopathology, ADHD symptoms, and conduct problems and found that these symptoms are inversely related to gray matter volumes across every region in the brain. This is consistent with prior univariate analyses demonstrating that general psychopathology is associated with a nearly global pattern of smaller gray matter volumes. Specifically, Durham et al. (2021) tested the association between psychopathology and each brain volume individually using the same ABCD Study dataset (*N* = 9,607) and the same psychopathology dimensions (general psychopathology, internalizing symptoms, ADHD symptoms, and conduct problems) (Durham et al., 2021). Following correction for multiple comparisons, this study found that greater general psychopathology scores were associated with smaller gray matter volumes in 54 out of 68 cortical regions and all 19 subcortical regions tested (Durham et al., 2021). This global pattern has also been shown in an independent sample of 1,394 children, adolescents, and young adults. Using data from the Philadelphia Neurodevelopmental Cohort, psychopathology dimensions (general psychopathology, anxious-misery, psychosis, behavioral, and fear) were related to structural covariance networks derived with non-negative matrix factorization (Kaczkurkin et al., 2019). Univariate analyses corrected for multiple comparisons demonstrated that general psychopathology was associated with smaller gray matter volumes in all brain regions (Kaczkurkin et al., 2019). This demonstrates that multivariate and univariate approaches may show concordance in brain-behavior relationships, at least in the context of the leading component found in multivariate approaches, and that the pattern of these results replicates across datasets.

The advantage of multivariate methods over univariate approaches is the ability to simultaneously examine associations between two sets of correlated variables, which allows us to account for multiple comparisons in a single model and enables us to capture complex interrelationships between variables not readily revealed with univariate analyses. Thus, while the leading component of CCA, PLSC, and PLSR all identified the same brain-behavior pattern found with univariate analyses, the benefit of multivariate approaches comes from the ability to extract multiple components, revealing additional brain-behavior associations. This is also where we start to see divergence between the methods in terms of interpretation. CCA, PLSC, and PLSR all extracted a second component, but the relative contributions of psychopathology symptoms to this component varied. The second component was strongly influenced by ADHD symptoms, but there was variability in the contribution of other symptoms to this profile depending on the method used. The second component reflected ADHD symptoms and internalizing symptoms in CCA, only ADHD symptoms in PLSC, and ADHD symptoms and general psychopathology symptoms in PLSR. Not surprisingly, there were both common and divergent patterns revealed in the brain regions implicated. Across approaches, we see similar patterns for the caudal middle frontal, inferior parietal, superior, middle, and inferior temporal, and posterior cingulate regions, likely due to the common contribution of ADHD symptoms to this component, which is consistent with prior research implicating these regions in ADHD (Sowell et al., 2003; Nakao et al., 2011; Yap et al., 2021). At the same time, a number of brain regions showed opposite patterns across the three methods for this component, which may be expected given the variations in the psychopathology symptoms represented. Thus, these results demonstrate that the relative contribution of psychopathology symptoms extracted from the data will have an impact the brain regions implicated, and what is extracted will vary based on the method chosen.

PLSR was the only multivariate method that extracted a third significant component. The third component yielded by PLSR primarily consisted of general psychopathology symptoms with conduct problems also contributing to this component. Interestingly, ADHD symptoms showed an inverse pattern, with ADHD symptoms *not* being strongly associated with the third component of PLSR. This component showed the opposite pattern seen previously in the second component in terms of the brain: we see *smaller* brain regions in the caudal middle frontal, inferior parietal, superior, middle, and inferior temporal, and posterior cingulate regions. This might be expected as ADHD was a strong driver of the second component and now ADHD is contributing in the opposite direction in this third component. Other brain regions implicated in the third component in PLSR were unique to this component, namely patterns in the frontal and lateral occipital regions. Thus, this illustrates that the number of components extracted will vary by multivariate method. The purpose in showing the findings of these three methods alongside each other is to illustrate that choice of approach matters and can greatly influence the brain-behavior relationships that will be found and the interpretation of those patterns.

As one concrete example to underscore how each approach leads to different conclusions about brain-behavior relationships, we can consider the caudal-middle frontal region, an area that was implicated in the second component of all three methods (labeled as 1 in Figures 2–4). If we had taken a CCA approach, we would conclude that larger volume in the caudal-middle frontal region is associated with ADHD and internalizing symptoms. However, if we had chosen PLSC, we would conclude that larger volume in the caudal-middle frontal region is associated with ADHD symptoms (and not at all with internalizing symptoms). And finally, had we used PLSR, we would conclude that larger volume in the caudal-middle frontal region is associated with all four psychopathology symptom dimensions (internalizing, conduct problems, ADHD, and general psychopathology) but with ADHD and general psychopathology making the greatest contributions. While ADHD is a common denominator amongst the results in the example of the caudal-middle frontal region, the relative contributions of other psychopathology dimensions differ entirely based on the approach chosen. The take home point is that not enough researchers are aware of the differences in interpretation that can arise between different multivariate approaches; thus, our goal is to make this point more salient for those in our field.

The divergence between the results of these three multivariate approaches is related to the distinctive underlying assumptions of these methods. The purpose of this article is not to provide a comprehensive tutorial on the differences between multivariate methods – for methodical comparisons, see prior discussions (Helmer et al., 2021; McIntosh, 2021; Mihalik et al., 2022); instead, the purpose of this study is to increase awareness regarding the divergence in interpretation when using different methods and to encourage thoughtful consideration when choosing an approach. That being said, here we provide a brief overview of some of the primary differences between the methods that should be taken into consideration when choosing an approach. As discussed in the introduction and methods, CCA results in components that standardize the covariance and maximize the correlation between the two sets of data; PLSC extracts components that account for the maximum covariance between the two sets; and PLSR results in components that reflect the predictive relationship of one set of data to another (McIntosh, 2021; Mihalik et al., 2022). Thus, while similar, these approaches are informed and driven by different philosophies around the nature of the data being analyzed (McIntosh, 2021). The underlying differences between CCA and PLS more broadly have important implications for neuroimaging research. As McIntosh (2021) notes in their comparison of CCA and PLS, CCA focuses on the unique contributions variables in one set make to the prediction of variables in the second set by removing redundancies, while PLS allows redundancies to remain with the assumption that these redundancies are a meaningful feature of the data. Thus, in the case of neuroimaging research, CCA will extract the unique contributions of brain regions in the association with symptoms while PLS tells us how the collective contribution of brain regions relate to symptoms (McIntosh, 2021). In other words, CCA is assuming the covariance structure between input variables is noise and should be removed to reflect the unique contributions between variable sets. In contrast, PLS assumes that the covariance between variables is meaningful signal that should be retained. Thus, when it comes to the question of which specific method to use, the decision should be informed by the goals of the particular research study and by whether the researcher intends to evaluate the collective contribution or the unique contributions of different variables within multivariate relationships. Importantly, there is no “right” answer – all methods are mathematically correct in their own way, they simply have different goals and assumptions. If the researcher is interested in the unique contributions brain regions have on psychopathology symptoms, then CCA would be appropriate. If the researcher believes that the covariance between brain regions may be meaningful, then PLS is appropriate. Regardless of which method is used, a clear justification needs to be made for choosing one over the other, which is not current practice in neuroimaging publications.

Beyond their underlying assumptions, multivariate approaches also have important limitations. While the limitations of each approach are beyond the scope of this paper, here we note several key issues to consider when using these approaches. For a more in-depth discussion of the limitations of multivariate models, we refer the reader to comprehensive discussions on these topics (Helmer et al., 2021; McIntosh, 2021; Mihalik et al., 2022). The first consideration is that it can be difficult to directly compare the reliability of different approaches to each other. It is well established that PLS-type approaches are biased toward the first principle component of the data which can create the appearance of greater reliability or stability between split half samples compared to CCA (Hastie et al., 2009; Helmer et al., 2021). Sample size is an additional critical consideration when using multivariate approaches. Recent work suggests that at least 50 samples per feature (i.e., input variables) are needed to obtain stable estimates for CCA (Helmer et al., 2021), which is much greater than that used by most published CCA studies and is especially relevant to brain-behavior studies where high dimensional brain features may be of interest. Importantly, the need for many samples per feature is not specific to CCA – all multivariate methods can be unreliable when there are insufficient sample sizes, illustrating the need for very large datasets to achieve reliable brain-behavior results (Bzdok and Yeo, 2017; Schulz et al., 2020). Additionally, strong correlations among neurobiological variables need to be considered. Given the expected high correlations among brain regions, a few variance inflation factor values exceeded the standard cutoff of 10 (James et al., 2013) in the current study, which could impact the CCA results. However, multicollinearity has been shown to be less problematic in large samples compared to smaller ones (James et al., 2013). In contrast, the PLS methods are largely robust to multicollinearity (Palermo et al., 2009; Wondola et al., 2020). Finally, it is important to note that the loadings of particular brain regions and psychopathology factors on the latent variables yielded by CCA/PLSC/PLSR analysis may vary slightly across different algorithms and software platforms. Thus, in addition to choosing an approach based on an understanding of their underlying assumptions, researchers must also be aware of the limitations of these methods.

In sum, multivariate examination of brain-behavior relationships has increased in popularity (Berman et al., 2014; Lin et al., 2018; Moser et al., 2018; Rodrigue et al., 2018; Stout et al., 2018; Kebets et al., 2019; Mihalik et al., 2019; Supekar et al., 2019; Kaczkurkin et al., 2020; Bolt et al., 2021); however, many assume these methods to be interchangeable and the majority of studies adopting one of these multivariate approaches do not provide a clear reasoning or justification for method selection. Here we illustrate the congruence (and lack thereof) of three different multivariate approaches (CCA, PLSC, and PLSR) for investigating brain-behavior associations in a large sample of children. We provide the following take-aways or conclusions. First, the present findings demonstrate how these methods produce both convergent and divergent interpretations around brain-behavior relationships as a result of different underlying assumptions. Second, it is critical for those interested in studying brain-behavior relationships to make an informed *a priori* decision about which method they will use and provide a justification for the choice of that method. Third, the choice of approach will depend on the goals of the study, whether one considers the covariance between variables to be noise (CCA) or signal (PLS), and whether one wants to identify correlational relationships between two datasets (CCA, PLSC) or predict one dataset using another dataset (PLSR). Fourth and finally, this study highlights and exemplifies the importance of carefully considering the underlying assumptions and limitations when choosing a multivariate approach to delineate brain-behavior relationships.

## Data availability statement

Data from the ABCD Study is publicly available through the National Institute of Mental Health Data Archive (https://nda.nih.gov/abcd). The code and corresponding descriptions of the statistical procedures for the current project can be found at https://github.com/VU-BRAINS-lab/ABCD_PLSCCA_Vol.

## Ethics statement

Use of this deidentified, publicly available dataset was reviewed and approved by the Vanderbilt University Institutional Review Board. Written informed consent to participate in this study was obtained by the ABCD Study researchers and provided by the participants’ legal guardian/next of kin.

## Author contributions

ED contributed to analyzing the data, writing the manuscript and supplement, making the tables and figures, and making revisions, KG contributed to coding data analysis scripts, analyzing data, and revising manuscript. AS and CC-I contributed to conception of study, coding data analysis scripts, analyzing data, and revising manuscript. GR, HJ, and XD contributed to writing of the manuscript. RD contributed to data preparation and revising of the manuscript. TM, MB, and DB provided conceptual and statistical consultation and contributed to revising the manuscript. BL provided conceptual and statistical consultation, contributed to the revising of the manuscript, and contributed funding to support this project. AK provided conceptual consultation, contributed to data analyses, contributed to the writing of the manuscript, and served as the primary mentor to ED, GR, HJ, RD, and XD. All authors provided critical feedback and helped shape the research, analysis and manuscript.

## Funding

This research was supported by grants UG3DA045251 (BBL) from the National Institute on Drug Abuse, R01MH098098 (BL), R01MH117014 (TM), R00MH117274 (AK), and T32MH18921 (ED is a trainee on this grant) from the National Institute of Mental Health, UL1TR000430 (BL) and UL1TR000445 (BL) from the National Center for Advancing Translational Sciences, a Young Investigator Grant from the Brain & Behavior Research Foundation (AK), a Sloan Research Fellowship (AK), a Seeding Success grant from Vanderbilt University (AK), and the Lifespan Brain Institute of the University of Pennsylvania and the Children’s Hospital of Philadelphia (TM). This material is also based upon work supported by the National Science Foundation Graduate Research Fellowship Program under Grant No. 1937963 (GER). Any opinions, findings, and conclusions or recommendations expressed in this material are those of the authors and do not necessarily reflect the views of the National Science Foundation. The ABCD Study® is supported by the National Institutes of Health and additional federal partners under award numbers U01DA041048, U01DA050989, U01DA051016, U01DA041022, U01DA051018, U01DA051037, U01DA050987, U01DA041174, U01DA041106, U01DA041117, U01DA041028, U01DA041134, U01DA050988, U01DA051039, U01DA041156, U01DA041025, U01DA041120, U01DA051038, U01DA041148, U01DA041093, U01DA041089, U24DA041123, and U24DA041147. A full list of supporters is available at https://abcdstudy.org/federal-partners.html.

## Conflict of interest

The authors declare that the research was conducted in the absence of any commercial or financial relationships that could be construed as a potential conflict of interest.

## Publisher’s note

All claims expressed in this article are solely those of the authors and do not necessarily represent those of their affiliated organizations, or those of the publisher, the editors and the reviewers. Any product that may be evaluated in this article, or claim that may be made by its manufacturer, is not guaranteed or endorsed by the publisher.

## Footnotes

^1^ https://www.statmodel.com/

^2^ https://scikit-learn.org/stable/index.html
