# Supplementary material for: Multivariate analytical approaches for investigating brain-behavior relationships
Source: Front Neurosci. 2023 Jul 31;17:1175690. doi: 10.3389/fnins.2023.1175690 (PMC10423877; doi:10.3389/fnins.2023.1175690)
Supplement: Supplementary file 1 [file Data_Sheet_1.docx]

Supplementary Material

Multivariate Analytical Approaches for Investigating Brain-Behavior Relationships

**E. Leighton Durham†,^1^ Karam Ghanem†**,**^2^ Andrew J. Stier,^3^ Carlos Cardenas-Iniguez,^4^ Gabrielle E. Reimann,^1^ Hee Jung Jeong,^1^ Randolph M. Dupont,^1^ Xiaoyu Dong,^1^ Tyler M. Moore,^5^ Marc G. Berman,^3,6^ Benjamin B. Lahey,^7^ Danilo Bzdok,^2^ and Antonia N. Kaczkurkin^1*^**

**† First authorship:** These authors share first authorship

*** Correspondence:** Antonia N. Kaczkurkin: [antonia.kaczkurkin@vanderbilt.edu](mailto:antonia.kaczkurkin@vanderbilt.edu)

# Supplementary information

## Generalizability of the ABCD sample

Procedures for the sampling and recruitment of participants for the ABCD Study is detailed elsewhere (Garavan et al., 2018). To summarize, there are 21 data collection sites across the country. ABCD researchers conducted probability sampling of schools that were within each of the catchment areas. Children that were eligible for the ABCD Study from each of the schools were recruited for study participation. Several sociodemographic variables were considered during participant recruitment including age, gender, socio-economic status, race/ethnicity, and urbanicity. The particular target numbers for these various demographic considerations were based on the American Community Survey (ACS) (an annual U.S. Census Bureau survey) and data on school enrollment from the National Center for Education Statistics. While the ABCD Study sites themselves are not exactly representative of the general U.S. population, the recruitment process was consistent across each site and unbiased.

## **Exclusions for primary PLS and CCA analyses**

In the current project, 1,184 participants were excluded due to having missing data on variables included in analysis or to failure to pass data quality assurance measures. More specifically, participants were excluded if they were missing data for the following variables and measures: Child Behavior Checklist (CBCL), post-stratification weight (PSWEIGHT), sex, race/ethnicity, parent education level, and imaging quality assurance variables (imgincl_t1w_include; mrif_score). Participants were also excluded if their scores on imaging quality assurance measures were outside of the acceptable range (imgincl_t1w_include= 0; mrif_score values of 0, 3, 4; fsqc_qu_motion > 1; fsqc_qu_ pialover > 1; fsqc_qu_wmunder > 1; and fsqc_qu_inhomogeneity > 1). An additional 1,665 same-family participants were randomly excluded to control for non-independence between twins and siblings. The final sample size used for primary analysis was N = 9,027.

# 1.3 Bifactor Modeling and the Child Behavior Checklist

The bifactor modeling procedures are thoroughly detailed in Moore et al. (2020) (Moore et al., 2020). To summarize, exploratory analyses reduced the number of included Child Behavior Checklist (CBCL) items to those that best reflect psychopathology at ages 9 and 10 years old. Items that were eliminated were excluded for a variety of reasons. Some of the primary reasons for item exclusion were as follows: not reflective of psychopathology (i.e., constipation or fingernail biting), not age-appropriate (i.e., substance use items), low endorsement, and similar to another item (composites of similar items were created). Following exploratory analyses with one random half of the CBCL data, a confirmatory bifactor analysis defined a general psychopathology factor, which is reflective of the symptoms shared across all participants, as well as specific internalizing symptoms, conduct problems, and ADHD symptoms factors. All four factors are orthogonal, and each included CBCL item loads onto both the general factor, as well as one of the three specific factors (internalizing, conduct, or ADHD). As an example, the item, “can’t sit still, restless, or hyperactive” loads onto the general and ADHD symptoms factors. The item, “cruel to animals”, loads onto the general and conduct problems factors. Finally, the item, “feels has to be perfect”, loads onto the general and internalizing symptoms factors.

# 1.4 Image Acquisition, Processing, and Quality Assurance

The ABCD Data Analysis and Informatics Center (DAIC), as well as the ABCD Imaging Acquisition Workgroup developed the scanning protocol for the ABCD study. The protocol was harmonized for all scanner platforms. Scanning occurred in one or two sessions and included T1- and T2-weighted images of brain structure. As previously detailed (Casey et al., 2018), imaging data collection occurred at 21 sites using a number of models of 3 tesla (3T) scanners from three different vendors: Siemens, General Electric, and Phillips. The specific scanner models used for data collection were General Electric Discovery MR750, Siemens Prisma, Siemens Prisma Fit, Phillips Achieva dStream, and Phillips Ingenia. More details on the imaging parameters are as follows: TR (repetition time) 2400 to 2500 ms; TE (echo time) 2 to 2.9 ms; FOV (field of view) 256 × 240 to 256; FOV phase of 93.75% to 100%; matrix 256 × 256; 176 to 225 slices; TI (inversion delay) 1060 ms; flip angle of 8°; voxel resolution of 1×1×1×mm; total acquisition time from 5 minutes 38 seconds to 7 minutes 12 seconds.

Thorough descriptions of the processing and analysis of brain data can be found elsewhere (Hagler et al., 2019). To summarize, DAIC performed centralized processing and analysis of the structural data using a collection of processing steps within the Multi-Modal Processing Stream (MMPS), which is a software package developed and maintained at the Center for Multimodal Imaging and Genetic (CMIG) at the University of California, San Diego (UCSD). Briefly, this pipeline included: 1) preprocessing (correction for gradient nonlinearity distortions, intensity scaling and inhomogeneity correction, registration to an averaged reference brain in standard space, and manual quality control (QC)); 2) brain segmentation (﻿cortical surface reconstruction and subcortical segmentation performed based on automated, atlas-based, segmentation procedures in FreeSurfer v5.3); 3) derivation of morphometric measures (calculation of average volume in each cortical parcel of the standard FreeSurfer Desikan parcellation scheme (Desikan et al., 2006) and in each subcortical region (Fischl et al., 2002)); and finally, 4) post-processing QC (manual review by trained technicians for ﻿motion, intensity inhomogeneity, white matter underestimation, pial overestimation, and magnetic susceptibility artifact).

**1.5 References**

Casey, B. J., Cannonier, T., Conley, M. I., Cohen, A. O., Barch, D. M., Heitzeg, M. M., et al. (2018). The Adolescent Brain Cognitive Development (ABCD) study: Imaging acquisition across 21 sites. *Dev. Cogn. Neurosci.* 32, 43–54. doi: 10.1016/j.dcn.2018.03.001.

Desikan, R. S., Ségonne, F., Fischl, B., Quinn, B. T., Dickerson, B. C., Blacker, D., et al. (2006). An automated labeling system for subdividing the human cerebral cortex on MRI scans into gyral based regions of interest. *Neuroimage* 31, 968–980. doi: 10.1016/j.neuroimage.2006.01.021.

Fischl, B., Salat, D. H., Busa, E., Albert, M., Dieterich, M., Haselgrove, C., et al. (2002). Whole brain segmentation: Automated labeling of neuroanatomical structures in the human brain. *Neuron* 33, 341–355. doi: 10.1016/S0896-6273(02)00569-X.

Garavan, H., Bartsch, H., Conway, K., Decastro, A., Goldstein, R. Z., Heeringa, S., et al. (2018). Recruiting the ABCD sample: Design considerations and procedures. *Dev. Cogn. Neurosci.* 32, 16–22. doi: 10.1016/j.dcn.2018.04.004.

Hagler, D. J., Hatton, S., Cornejo, M. D., Makowski, C., Fair, D. A., Dick, A. S., et al. (2019). Image processing and analysis methods for the Adolescent Brain Cognitive Development Study. *Neuroimage* 202. doi: 10.1016/j.neuroimage.2019.116091.

Moore, T. M., Kaczkurkin, A. N., Durham, E. L., Jeong, H. J., McDowell, M. G., Dupont, R. M., et al. (2020). Criterion Validity and Relationships between Alternative Hierarchical Dimensional Models of General and Specific Psychopathology. *J. Abnorm. Psychol.* 129, 677–688.

# Supplementary Tables

**2.1** **Table S1. *Variance inflation factor values for all variables included in CCA, PLSC,***

***and PLSR analyses.***

| Psychopathology Dimension | VIF |  |  |
| --- | --- | --- | --- |
| General Psychopathology | 1.09 |  | |
| Conduct Problems | 1.12 |  | |
| ADHD Symptoms | 1.02 |  | |
| Internalizing Symptoms | 1.12 |  | |
| Brain Region Name | VIF |  |  |
|  |  |  |  |
| Brain stem | 3.66 |  |  |
| Left accumbens area | 2.53 |  |  |
| Left amygdala | 2.45 |  |  |
| Left banks of superior temporal sulcus | 1.68 |  |  |
| Left caudal anterior cingulate | 1.75 |  |  |
| Left caudal middle frontal | 2.08 |  |  |
| Left caudate | 8.20 |  |  |
| Left cerebellum cortex | 10.05 |  |  |
| Left cuneus | 3.20 |  |  |
| Left entorhinal | 1.72 |  |  |
| Left frontal pole | 1.50 |  |  |
| Left fusiform | 2.39 |  |  |
| Left hippocampus | 3.95 |  |  |
| Left inferior parietal | 3.04 |  |  |
| Left inferior temporal | 2.75 |  |  |
| Left insula | 4.15 |  |  |
| Left isthmus cingulate | 1.90 |  |  |
| Left lateral occipital | 2.63 |  |  |
| Left lateral orbitofrontal | 4.04 |  |  |
| Left lingual | 3.22 |  |  |
| Left medial orbitofrontal | 2.22 |  |  |
| Left middle temporal | 3.13 |  |  |
| Left pallidum | 3.14 |  |  |
| Left paracentral | 1.87 |  |  |
| Left parahippocampal | 1.87 |  |  |
| Left pars opercularis | 1.87 |  |  |
| Left pars orbitalis | 1.95 |  |  |
| Left pars triangularis | 2.14 |  |  |
| Left pericalcarine | 5.38 |  |  |
| Left postcentral | 2.58 |  |  |
| Left posterior cingulate | 1.86 |  |  |
| Left precentral | 3.16 |  |  |
| Left precuneus | 3.53 |  |  |
| Left putamen | 11.72 |  |  |
| Left rostral anterior cingulate | 2.09 |  |  |
| Left rostral middle frontal | 3.29 |  |  |
| Left superior frontal | 4.58 |  |  |
| Left superior parietal | 3.12 |  |  |
| Left superior temporal | 3.54 |  |  |
| Left supramarginal | 2.72 |  |  |
| Left temporal pole | 1.63 |  |  |
| Left thalamus proper | 5.46 |  |  |
| Left transverse temporal | 1.99 |  |  |
| Left ventral diencephalon | 7.17 |  |  |
| Right accumbens area | 2.46 |  |  |
| Right amygdala | 2.82 |  |  |
| Right banks of superior temporal sulcus | 1.79 |  |  |
| Right caudal anterior cingulate | 2.02 |  |  |
| Right caudal middle frontal | 2.10 |  |  |
| Right caudate | 8.79 |  |  |
| Right cerebellum cortex | 9.90 |  |  |
| Right cuneus | 3.16 |  |  |
| Right entorhinal | 1.63 |  |  |
| Right frontal pole | 1.45 |  |  |
| Right fusiform | 2.44 |  |  |
| Right hippocampus | 3.71 |  |  |
| Right inferior parietal | 3.09 |  |  |
| Right inferior temporal | 2.63 |  |  |
| Right insula | 3.56 |  |  |
| Right isthmus cingulate | 1.86 |  |  |
| Right lateral occipital | 2.64 |  |  |
| Right lateral orbitofrontal | 4.12 |  |  |
| Right lingual | 3.23 |  |  |
| Right medial orbitofrontal | 2.51 |  |  |
| Right middle temporal | 3.35 |  |  |
| Right pallidum | 3.25 |  |  |
| Right paracentral | 1.86 |  |  |
| Right parahippocampal | 1.70 |  |  |
| Right pars opercularis | 1.88 |  |  |
| Right pars orbitalis | 1.94 |  |  |
| Right pars triangularis | 2.05 |  |  |
| Right pericalcarine | 5.03 |  |  |
| Right postcentral | 2.53 |  |  |
| Right posterior cingulate | 1.96 |  |  |
| Right precentral | 3.37 |  |  |
| Right precuneus | 3.63 |  |  |
| Right putamen | 11.21 |  |  |
| Right rostral anterior cingulate | 1.90 |  |  |
| Right rostral middle frontal | 3.41 |  |  |
| Right superior frontal | 4.61 |  |  |
| Right superior parietal | 3.36 |  |  |
| Right superior temporal | 3.70 |  |  |
| Right supramarginal | 2.78 |  |  |
| Right temporal pole | 1.65 |  |  |
| Right thalamus proper | 5.26 |  |  |
| Right transverse temporal | 2.05 |  |  |
| Right ventral diencephalon | 7.23 |  |  |

*Note.* Regions are listed in alphabetical order by region name. VIF refers to variance inflation factor.

# 2.2 Table S2. *Psychopathology loadings on the two significant components yielded by CCA analysis examining the relationship between regional GMV and psychopathology dimensions*

| Psychopathology Dimension | Component 1 | Component 2 |
| --- | --- | --- |
| General Psychopathology | 0.766 | -0.280 |
| Conduct Problems | 0.494 | -0.197 |
| ADHD Symptoms | 0.394 | 0.900 |
| Internalizing Symptoms | -0.149 | 0.403 |

*Note.* N = 9,027. Only results for significant components are presented. Components are in order of significance.

#

# 2.3 Table S3. *Brain loadings on the two significant components yielded by CCA analysis examining the relationship between regional GMV and psychopathology dimensions*

| Brain Region Name | Component 1 | Component 2 |
| --- | --- | --- |
| Brain stem | -0.698 | -0.084 |
| Left accumbens area | -0.480 | -0.222 |
| Left amygdala | -0.375 | 0.040 |
| Left banks of superior temporal sulcus | -0.469 | 0.213 |
| Left caudal anterior cingulate | -0.452 | -0.244 |
| Left caudal middle frontal | -0.499 | 0.050 |
| Left caudate | -0.570 | -0.211 |
| Left cerebellum cortex | -0.684 | 0.128 |
| Left cuneus | -0.277 | -0.254 |
| Left entorhinal | -0.321 | -0.124 |
| Left frontal pole | -0.193 | -0.054 |
| Left fusiform | -0.623 | 0.029 |
| Left hippocampus | -0.654 | 0.324 |
| Left inferior parietal | -0.612 | 0.179 |
| Left inferior temporal | -0.536 | 0.310 |
| Left insula | -0.705 | -0.027 |
| Left isthmus cingulate | -0.530 | 0.009 |
| Left lateral occipital | -0.508 | -0.235 |
| Left lateral orbitofrontal | -0.658 | -0.129 |
| Left lingual | -0.603 | -0.093 |
| Left medial orbitofrontal | -0.379 | -0.216 |
| Left middle temporal | -0.658 | 0.247 |
| Left pallidum | -0.452 | -0.327 |
| Left paracentral | -0.514 | -0.124 |
| Left parahippocampal | -0.483 | -0.061 |
| Left pars opercularis | -0.320 | -0.049 |
| Left pars orbitalis | -0.463 | -0.003 |
| Left pars triangularis | -0.453 | -0.458 |
| Left pericalcarine | -0.265 | -0.143 |
| Left postcentral | -0.640 | -0.086 |
| Left posterior cingulate | -0.561 | 0.137 |
| Left precentral | -0.929 | -0.003 |
| Left precuneus | -0.654 | -0.114 |
| Left putamen | -0.579 | -0.423 |
| Left rostral anterior cingulate | -0.600 | -0.184 |
| Left rostral middle frontal | -0.674 | -0.143 |
| Left superior frontal | -0.729 | -0.056 |
| Left superior parietal | -0.537 | -0.171 |
| Left superior temporal | -0.739 | 0.070 |
| Left supramarginal | -0.735 | 0.175 |
| Left temporal pole | -0.397 | 0.211 |
| Left thalamus proper | -0.686 | 0.006 |
| Left transverse temporal | -0.382 | -0.168 |
| Left ventral diencephalon | -0.659 | 0.042 |
| Right accumbens area | -0.615 | -0.154 |
| Right amygdala | -0.457 | 0.121 |
| Right banks of superior temporal sulcus | -0.348 | 0.226 |
| Right caudal anterior cingulate | -0.588 | -0.178 |
| Right caudal middle frontal | -0.445 | 0.067 |
| Right caudate | -0.533 | -0.320 |
| Right cerebellum cortex | -0.628 | 0.183 |
| Right cuneus | -0.349 | -0.124 |
| Right entorhinal | -0.483 | -0.013 |
| Right frontal pole | -0.299 | -0.170 |
| Right fusiform | -0.709 | 0.023 |
| Right hippocampus | -0.596 | 0.145 |
| Right inferior parietal | -0.633 | 0.179 |
| Right inferior temporal | -0.656 | 0.191 |
| Right insula | -0.700 | -0.128 |
| Right isthmus cingulate | -0.435 | -0.023 |
| Right lateral occipital | -0.493 | -0.143 |
| Right lateral orbitofrontal | -0.687 | -0.059 |
| Right lingual | -0.410 | -0.253 |
| Right medial orbitofrontal | -0.545 | -0.147 |
| Right middle temporal | -0.792 | 0.338 |
| Right pallidum | -0.423 | -0.269 |
| Right paracentral | -0.609 | -0.285 |
| Right parahippocampal | -0.399 | 0.071 |
| Right pars opercularis | -0.240 | 0.079 |
| Right pars orbitalis | -0.565 | -0.399 |
| Right pars triangularis | -0.282 | -0.190 |
| Right pericalcarine | -0.328 | -0.113 |
| Right postcentral | -0.688 | -0.084 |
| Right posterior cingulate | -0.649 | 0.152 |
| Right precentral | -0.854 | 0.010 |
| Right precuneus | -0.561 | -0.088 |
| Right putamen | -0.607 | -0.520 |
| Right rostral anterior cingulate | -0.570 | -0.146 |
| Right rostral middle frontal | -0.639 | -0.022 |
| Right superior frontal | -0.665 | -0.025 |
| Right superior parietal | -0.472 | -0.170 |
| Right superior temporal | -0.609 | 0.092 |
| Right supramarginal | -0.685 | -0.002 |
| Right temporal pole | -0.388 | -0.042 |
| Right thalamus proper | -0.559 | 0.030 |
| Right transverse temporal | -0.423 | 0.029 |
| Right ventral diencephalon | -0.626 | -0.011 |

*Note.* N = 9,027. Loadings are listed in alphabetical order by region name. Only results for significant components are presented. Components are in order of significance.

## 2.4 Table S4. *Psychopathology loadings on the two significant components yielded by PLSC analysis examining the relationship between regional GMV and psychopathology dimensions*

| Psychopathology Dimension | Component 1 | Component 2 |
| --- | --- | --- |
| General Psychopathology | 0.701 | -0.283 |
| Conduct Problems | 0.554 | -0.391 |
| ADHD Symptoms | 0.408 | 0.881 |
| Internalizing Symptoms | -0.199 | -0.041 |

*Note.* N = 9,027. Only results for significant components are presented. Components are in order of significance.

## 2.5 Table S5. *Brain loadings on the two significant components yielded by PLSC analysis examining the relationship between regional GMV and psychopathology dimensions*

| Brain Region Name | Component 1 | Component 2 |
| --- | --- | --- |
| Brain stem | -0.117 | -0.009 |
| Left accumbens area | -0.113 | -0.047 |
| Left amygdala | -0.106 | 0.110 |
| Left banks of superior temporal sulcus | -0.086 | 0.210 |
| Left caudal anterior cingulate | -0.078 | -0.038 |
| Left caudal middle frontal | -0.102 | 0.072 |
| Left caudate | -0.103 | -0.133 |
| Left cerebellum cortex | -0.103 | 0.094 |
| Left cuneus | -0.072 | -0.247 |
| Left entorhinal | -0.063 | -0.012 |
| Left frontal pole | -0.077 | 0.003 |
| Left fusiform | -0.121 | 0.053 |
| Left hippocampus | -0.130 | 0.096 |
| Left inferior parietal | -0.106 | 0.192 |
| Left inferior temporal | -0.117 | 0.197 |
| Left insula | -0.132 | -0.072 |
| Left isthmus cingulate | -0.104 | -0.042 |
| Left lateral occipital | -0.105 | -0.116 |
| Left lateral orbitofrontal | -0.140 | -0.102 |
| Left lingual | -0.092 | -0.151 |
| Left medial orbitofrontal | -0.120 | -0.036 |
| Left middle temporal | -0.121 | 0.256 |
| Left pallidum | -0.105 | -0.125 |
| Left paracentral | -0.104 | 0.008 |
| Left parahippocampal | -0.081 | 0.040 |
| Left pars opercularis | -0.087 | -0.148 |
| Left pars orbitalis | -0.102 | -0.129 |
| Left pars triangularis | -0.084 | -0.286 |
| Left pericalcarine | -0.069 | -0.228 |
| Left postcentral | -0.123 | 0.098 |
| Left posterior cingulate | -0.110 | 0.069 |
| Left precentral | -0.127 | 0.082 |
| Left precuneus | -0.126 | -0.051 |
| Left putamen | -0.098 | -0.198 |
| Left rostral anterior cingulate | -0.112 | -0.034 |
| Left rostral middle frontal | -0.125 | -0.082 |
| Left superior frontal | -0.139 | 0.003 |
| Left superior parietal | -0.110 | -0.035 |
| Left superior temporal | -0.132 | 0.097 |
| Left supramarginal | -0.105 | 0.111 |
| Left temporal pole | -0.081 | 0.040 |
| Left thalamus proper | -0.136 | -0.042 |
| Left transverse temporal | -0.089 | -0.074 |
| Left ventral diencephalon | -0.133 | -0.001 |
| Right accumbens area | -0.113 | -0.019 |
| Right amygdala | -0.121 | 0.092 |
| Right banks of superior temporal sulcus | -0.096 | 0.271 |
| Right caudal anterior cingulate | -0.076 | -0.002 |
| Right caudal middle frontal | -0.101 | 0.077 |
| Right caudate | -0.109 | -0.141 |
| Right cerebellum cortex | -0.103 | 0.093 |
| Right cuneus | -0.080 | -0.224 |
| Right entorhinal | -0.059 | -0.006 |
| Right frontal pole | -0.075 | -0.034 |
| Right fusiform | -0.125 | 0.044 |
| Right hippocampus | -0.125 | 0.106 |
| Right inferior parietal | -0.111 | 0.193 |
| Right inferior temporal | -0.119 | 0.158 |
| Right insula | -0.126 | -0.084 |
| Right isthmus cingulate | -0.097 | -0.045 |
| Right lateral occipital | -0.108 | -0.136 |
| Right lateral orbitofrontal | -0.139 | -0.097 |
| Right lingual | -0.095 | -0.136 |
| Right medial orbitofrontal | -0.127 | -0.043 |
| Right middle temporal | -0.131 | 0.258 |
| Right pallidum | -0.106 | -0.124 |
| Right paracentral | -0.103 | -0.009 |
| Right parahippocampal | -0.083 | 0.053 |
| Right pars opercularis | -0.084 | -0.070 |
| Right pars orbitalis | -0.101 | -0.188 |
| Right pars triangularis | -0.078 | -0.231 |
| Right pericalcarine | -0.073 | -0.218 |
| Right postcentral | -0.123 | 0.109 |
| Right posterior cingulate | -0.112 | 0.102 |
| Right precentral | -0.123 | 0.077 |
| Right precuneus | -0.130 | -0.047 |
| Right putamen | -0.108 | -0.197 |
| Right rostral anterior cingulate | -0.094 | -0.011 |
| Right rostral middle frontal | -0.117 | -0.081 |
| Right superior frontal | -0.135 | 0.001 |
| Right superior parietal | -0.110 | -0.024 |
| Right superior temporal | -0.138 | 0.125 |
| Right supramarginal | -0.110 | 0.109 |
| Right temporal pole | -0.083 | -0.045 |
| Right thalamus proper | -0.135 | -0.024 |
| Right transverse temporal | -0.098 | -0.006 |
| Right ventral diencephalon | -0.133 | -0.011 |

*Note.* N = 9,027. Loadings are listed in alphabetical order by region name. Only results for significant components are presented. Components are in order of significance.

# 2.6 Table S6. *Psychopathology loadings on the three significant components yielded by PLSR analysis examining the relationship between regional GMV and psychopathology dimensions*

| Psychopathology Dimension | Component 1 | Component 2 | Component 3 |
| --- | --- | --- | --- |
| General Psychopathology | 0.015 | 0.065 | 0.040 |
| Conduct Problems | 0.011 | 0.022 | 0.031 |
| ADHD Symptoms | 0.010 | 0.071 | -0.040 |
| Internalizing Symptoms | -0.004 | 0.022 | -0.001 |

*Note.* N = 9,027. Only results for significant components are presented. Components are in order of significance.

# 2.7 Table S7. *Brain loadings on the three significant components yielded by PLSR analysis examining the relationship between regional GMV and psychopathology dimensions*

| Brain Region Name | Component 1 | Component 2 | Component 3 |
| --- | --- | --- | --- |
| Brain stem | -0.117 | -0.026 | -0.014 |
| Left accumbens area | -0.113 | -0.021 | 0.049 |
| Left amygdala | -0.106 | 0.216 | -0.049 |
| Left banks of superior temporal sulcus | -0.086 | 0.069 | -0.205 |
| Left caudal anterior cingulate | -0.078 | -0.036 | 0.025 |
| Left caudal middle frontal | -0.102 | 0.014 | -0.078 |
| Left caudate | -0.103 | -0.110 | 0.109 |
| Left cerebellum cortex | -0.103 | -0.034 | -0.128 |
| Left cuneus | -0.072 | 0.078 | 0.273 |
| Left entorhinal | -0.063 | -0.078 | 0.002 |
| Left frontal pole | -0.077 | 0.227 | 0.059 |
| Left fusiform | -0.121 | 0.001 | -0.054 |
| Left hippocampus | -0.130 | 0.185 | -0.058 |
| Left inferior parietal | -0.106 | 0.062 | -0.178 |
| Left inferior temporal | -0.117 | 0.177 | -0.163 |
| Left insula | -0.132 | -0.048 | 0.060 |
| Left isthmus cingulate | -0.104 | 0.053 | 0.050 |
| Left lateral occipital | -0.105 | 0.059 | 0.136 |
| Left lateral orbitofrontal | -0.140 | 0.034 | 0.109 |
| Left lingual | -0.092 | 0.019 | 0.154 |
| Left medial orbitofrontal | -0.120 | 0.175 | 0.086 |
| Left middle temporal | -0.121 | 0.161 | -0.228 |
| Left pallidum | -0.105 | -0.003 | 0.129 |
| Left paracentral | -0.104 | -0.144 | -0.028 |
| Left parahippocampal | -0.081 | -0.011 | -0.048 |
| Left pars opercularis | -0.087 | 0.188 | 0.184 |
| Left pars orbitalis | -0.102 | 0.014 | 0.139 |
| Left pars triangularis | -0.084 | 0.009 | 0.292 |
| Left pericalcarine | -0.069 | 0.078 | 0.249 |
| Left postcentral | -0.123 | -0.125 | -0.115 |
| Left posterior cingulate | -0.110 | 0.033 | -0.067 |
| Left precentral | -0.127 | -0.205 | -0.131 |
| Left precuneus | -0.126 | -0.003 | 0.057 |
| Left putamen | -0.098 | -0.251 | 0.155 |
| Left rostral anterior cingulate | -0.112 | -0.054 | 0.018 |
| Left rostral middle frontal | -0.125 | 0.019 | 0.091 |
| Left superior frontal | -0.139 | -0.050 | -0.008 |
| Left superior parietal | -0.110 | -0.067 | 0.031 |
| Left superior temporal | -0.132 | 0.079 | -0.083 |
| Left supramarginal | -0.105 | -0.121 | -0.140 |
| Left temporal pole | -0.081 | 0.071 | -0.017 |
| Left thalamus proper | -0.136 | 0.071 | 0.048 |
| Left transverse temporal | -0.089 | 0.044 | 0.079 |
| Left ventral diencephalon | -0.133 | 0.050 | 0.002 |
| Right accumbens area | -0.113 | -0.070 | 0.013 |
| Right amygdala | -0.121 | 0.211 | -0.034 |
| Right banks of superior temporal sulcus | -0.096 | 0.226 | -0.226 |
| Right caudal anterior cingulate | -0.076 | -0.256 | -0.052 |
| Right caudal middle frontal | -0.101 | 0.097 | -0.063 |
| Right caudate | -0.109 | -0.112 | 0.117 |
| Right cerebellum cortex | -0.103 | -0.021 | -0.125 |
| Right cuneus | -0.080 | 0.074 | 0.249 |
| Right entorhinal | -0.059 | -0.179 | -0.023 |
| Right frontal pole | -0.075 | 0.060 | 0.065 |
| Right fusiform | -0.125 | -0.034 | -0.054 |
| Right hippocampus | -0.125 | 0.205 | -0.062 |
| Right inferior parietal | -0.111 | 0.025 | -0.189 |
| Right inferior temporal | -0.119 | 0.035 | -0.151 |
| Right insula | -0.126 | -0.064 | 0.071 |
| Right isthmus cingulate | -0.097 | 0.150 | 0.070 |
| Right lateral occipital | -0.108 | 0.026 | 0.146 |
| Right lateral orbitofrontal | -0.139 | 0.014 | 0.098 |
| Right lingual | -0.095 | 0.075 | 0.153 |
| Right medial orbitofrontal | -0.127 | 0.098 | 0.072 |
| Right middle temporal | -0.131 | 0.156 | -0.232 |
| Right pallidum | -0.106 | -0.010 | 0.127 |
| Right paracentral | -0.103 | -0.264 | -0.033 |
| Right parahippocampal | -0.083 | 0.055 | -0.046 |
| Right pars opercularis | -0.084 | 0.289 | 0.128 |
| Right pars orbitalis | -0.101 | -0.109 | 0.173 |
| Right pars triangularis | -0.078 | 0.108 | 0.259 |
| Right pericalcarine | -0.073 | 0.092 | 0.241 |
| Right postcentral | -0.123 | -0.135 | -0.132 |
| Right posterior cingulate | -0.112 | -0.104 | -0.121 |
| Right precentral | -0.123 | -0.236 | -0.131 |
| Right precuneus | -0.130 | 0.042 | 0.059 |
| Right putamen | -0.108 | -0.246 | 0.156 |
| Right rostral anterior cingulate | -0.094 | -0.164 | -0.029 |
| Right rostral middle frontal | -0.117 | 0.011 | 0.088 |
| Right superior frontal | -0.135 | 0.028 | 0.011 |
| Right superior parietal | -0.110 | -0.049 | 0.022 |
| Right superior temporal | -0.138 | 0.162 | -0.093 |
| Right supramarginal | -0.110 | -0.055 | -0.125 |
| Right temporal pole | -0.083 | 0.039 | 0.062 |
| Right thalamus proper | -0.135 | 0.097 | 0.036 |
| Right transverse temporal | -0.098 | 0.127 | 0.028 |
| Right ventral diencephalon | -0.133 | 0.040 | 0.009 |

*Note.* N = 9,027. Loadings are listed in alphabetical order by region name. Only results for significant components are presented. Components are in order of significance.
